# Unveiling the Antihyperglycemic Potential of *Arctium lappa* L. (Asteraceae): Traditional Application, Phytochemistry, and Molecular Insights

**DOI:** 10.3390/foods15040794

**Published:** 2026-02-23

**Authors:** Amangul A. Uzbekova, Kaldanay K. Kozhanova, Gulnara Kadyrbayeva, Bayan I. Tursubekova, Meruyert Amantayeva, Moldir A. Zhandabayeva, Meruyert I. Tleubayeva, Ahmet Beyatli

**Affiliations:** 1 School of Pharmacy, S.D. Asfendiyarov Kazakh National Medical University, Almaty 050012, Kazakhstan; amangul.askarovna@mail.ru (A.A.U.); chilnara_k@mail.ru (G.K.); amantaeva.meruert@kaznmu.kz (M.A.); zhandabaeva.m@kaznmu.kz (M.A.Z.); tleubayeva.m@kaznmu.kz (M.I.T.); 2 Department of Chemistry and Pharmaceutical Engineering, Auezov University, Shymkent 160000, Kazakhstan; btursubekova@inbox.ru; 3 Department of Medicinal and Aromatic Plants, Hamidiye Vocational School of Health Services, University of Health Sciences, Istanbul 34668, Türkiye; 4 School of Agriculture, Food and Ecosystem Sciences, Faculty of Science, Food Science and Nutrition, The University of Melbourne, Melbourne, VIC 3004, Australia

**Keywords:** *Arctium lappa*, antidiabetic effect, AMPK, GLUT4, phenolic compounds

## Abstract

Diabetes mellitus is a chronic disease requiring multifunctional natural agents. *Arctium lappa* is traditionally used in Eastern and European medicine to address metabolic disorders. This comprehensive narrative review, conducted between 2000 and 2025 using international databases (Scopus, PubMed, Web of Science Core Collection, and Google Scholar), evaluates the species through its ethnomedicine, phytochemistry, preclinical evidence, and safety. The available evidence suggests that *A. lappa* exerts antidiabetic effects via multi-layered mechanisms, including AMPK activation, insulin signaling modulation, and increased GLUT4 translocation. Key bioactives (arctigenin, arctiin, and inulin) collectively improve insulin sensitivity and lipid metabolism. However, preclinical studies confirm these effects in animal models, while limited clinical data in non-diabetic cohorts focus on systemic inflammation. This highlights a significant gap in randomized controlled trials targeting glycemic control in diabetic populations. In this context, while *A. lappa* shows promise as a potential metabolic regulator; this evidence is currently derived primarily from in vitro and animal models. Systematic clinical trials are urgently required to establish glycemic efficacy in humans, validate its therapeutic potential, and determine the optimal dosage and safety profile. This review evaluates the multi-targeted biological potential of *A. lappa* to guide future research and evidence-based application.

## 1. Introduction

Diabetes mellitus (DM) is one of the most significant chronic non-communicable diseases of the 21st century. According to a global analysis by Zhou et al. (2024), the total number of people living with diabetes has exceeded 800 million [1,2]. This epidemiological surge is further supported by the International Diabetes Federation (IDF), which reports that over 537 million adults (aged 20–79) currently live with the condition, a figure projected to rise to 643 million by 2030 and 783 million by 2045. The disparity in these global estimates (800 million vs. 537 million) reflects differences in population definitions, with higher figures accounting for all age groups and undiagnosed cases globally. Regardless of the specific metric, the magnitude of the epidemic highlights the need for immediate measures, especially in low- and middle-income countries. The largest proportion is occupied by cases of type 2 DM, characterized by insulin resistance, impaired carbohydrate metabolism and systemic complications affecting the cardiovascular, nervous and renal systems [2].

Beyond the epidemiological burden, DM represents a profound socio-economic challenge. According to the International Diabetes Federation (IDF), the total global health expenditure related to diabetes has exceeded 966 billion US dollars annually and is projected to surpass 1 trillion by 2030. This figure primarily accounts for direct medical costs; however, when indirect costs, such as labor productivity losses due to disability and premature mortality, are included, the total economic burden is significantly higher [3]. The disease is associated with an increased risk of severe cardiovascular complications, chronic renal failure, neuropathy, and visual impairments, which significantly reduce patients’ quality of life and work productivity. Furthermore, the rising morbidity among younger populations is particularly alarming, highlighting the impact of obesity, physical inactivity, and poor nutrition as primary risk factors [4]. Consequently, DM is increasingly recognized as a global epidemic requiring comprehensive, interdisciplinary approaches for prevention and treatment.

Despite the wide arsenal of modern drugs, including biguanides [5], sodium–glucose transporter 2 (SGLT2) inhibitors and Glucagon-Like Peptide-1 (GLP-1) agonists [6], glycemic control and prevention of complications remain a difficult task [7], especially in patients with limited financial resources [8] and concomitant diseases.

Modern antidiabetic drugs, although they show a marked decrease in blood glucose levels in the short term, often lose their clinical efficacy over time. This decline is primarily attributed to the natural progression of the disease, specifically the gradual decline in pancreatic beta-cell function, as well as challenges related to long-term medication adherence, side effects, and the exacerbation of insulin resistance [9]. In addition, the high cost of innovative medicines limits their accessibility to a significant proportion of patients, especially in low- and middle-income countries, which exacerbates inequalities in treatment outcomes.

**Figure 1:**
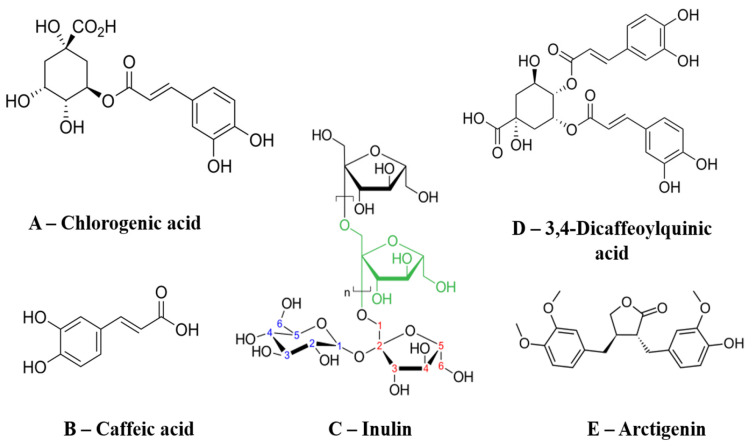
Chemical structures of the main bioactive compounds identified in *A. lappa*, including phenolic acids (A—chlorogenic acid and, B—caffeic acid), fructan polysaccharides (C—inulin), caffeoylquinic acid derivatives (D—dicaffeoylquinic acid) and lignans (E—arctigenin). (Source: [18,25]).

In this regard, there is increasing interest in natural sources of antihyperglycemic compounds. While often considered accessible, their therapeutic potential and safety profile are strictly dependent on rigorous extract standardization, comprehensive quality control, and the establishment of evidence-based dosing regimens [10]. In recent decades, there has been increased interest in the use of medicinal plants as alternatives or auxiliary agents in the treatment of diabetes. Many of these plants produce metabolites that modulate targets within the same pharmacological classes as conventional antidiabetic drugs, including natural components that act similarly to alpha-glucosidase inhibitors, insulin secretagogues, and insulin-sensitizing agents. Numerous studies confirm the effectiveness of these plant-derived compounds in lowering blood glucose levels, improving insulin sensitivity, and protecting pancreatic beta-cells from oxidative stress [11]. Specifically, plant extracts contain a wide range of biologically active compounds that exhibit biguanide-like metabolic effects, natural alpha-glucosidase inhibition, and thiazolidinedione-like insulin-sensitizing properties, as illustrated by the chemical structures in Figure 1.

According to modern reviews, more than 400 plant species, including *Allium sativum* (garlic), *Curcuma longa* (turmeric), and *Momordica charantia* (bitter melon), exhibit pronounced antihyperglycemic activity in vitro and in vivo [12,13]. In addition, clinical trials have shown that the use of phytocomplexes based on medicinal plants can lead to a statistically significant decrease in the level of glycated hemoglobin (HbA1c) in patients with type 2 DM [14]. Modern meta-analyses confirm that extracts of *Aloe vera*, *Trigonella foenum-graecum* (fenugreek) and *Nigella sativa* (black cumin) lead to a decrease in HbA1c by about 0.8–1.0% and improve lipid metabolism [15]. Other clinical and preclinical studies indicate a significant decrease in blood glucose levels and increased tissue sensitivity to insulin when using extracts of *Momordica charantia*, *Hibiscus sabdariffa* and *Zingiber officinale* (ginger) [16,17]. These data indicate the high prospects of phytotherapy as a safe and affordable approach to glycemic control, especially for patients with limited access to expensive medicines.

The researchers’ attention was attracted by *A. lappa*. It is widely used in traditional Chinese [18], Japanese and European medicine [19] as a remedy with detoxifying, antihyperglycemic properties [18,20,21]. The plant contains a rich complex of biologically active compounds, including inulin, lignans [21,22], flavonoids, phenolic acids, triterpenoids, and polysaccharides [21]. All of these compounds exhibit pronounced antidiabetic activity. Also, according to the results of numerous preclinical studies (in vitro and in vivo studies), they demonstrate a wide range of biological and pharmacological effects, including anti-inflammatory, antioxidant, antitumor, antidiabetic, antibacterial and neuroprotective effects. These properties are attributed to specific metabolites such as the lignans arctiin and arctigenin, inulin-type fructans, and phenolic acids, specifically caffeoylquinic acid derivatives (including chlorogenic acid and cynarine), as well as flavonoids like rutin and quercetin [22,23,24]. Figure 1 shows *A. lappa* compounds that have antihyperglycemic properties.

Due to its rich chemical composition and versatility, *A. lappa*, which is considered a promising source of natural compounds for the development of phytopreparations, is used for the prevention and complex therapy of DM and concomitant metabolic disorders [26,27].

While previous reviews have explored the general pharmacological properties of the Arctium genus, there remains a lack of an integrated synthesis focusing specifically on *A. lappa* within the modern context of diabetes management. This review uniquely addresses this gap by providing a comprehensive analysis of literature from the most recent decade (2000–2025); integrating previously fragmented mechanistic data, specifically regarding the AMPK/GLUT4 axis; synthesizing safety data alongside Human Equivalent Dose (HED) calculations; and framing the species within a functional-food-to-therapeutic-adjunct transition. Unlike earlier works, this review provides a prioritized framework for clinical translation and standardization of extracts.

Despite the fact that *A. lappa* is actively used in folk medicine to reduce blood sugar levels, the mechanisms of its Antihyperglycemic effect are still insufficiently systematized [18,28]. Modern studies show that its extracts are able to influence the expression of genes involved in regulating glucose homeostasis and improve insulin resistance [29,30], activating AMP-activated protein kinase (AMPK) [19] and stimulating glucose transporter type 4 (GLUT4) translocation, thus improving glucose utilization in peripheral tissues [19,31,32].

The purpose of this review is to summarize and analyze existing data on the antihyperglycemic potential of *A. lappa*, including its phytochemical composition, pharmacological research results, and molecular mechanisms of action. The review also addresses issues in safety and toxicological assessments, which are important for the prospects of developing phytopreparations based on this plant.

## 2. Methodology

A comprehensive narrative review was conducted across international databases (Scopus, PubMed, Web of Science, and Google Scholar) for the literature published between 2000 and 2025. The final search update and screening were completed in December 2025. The search utilized specific keywords and Boolean operators: (“*Arctium lappa*” OR “Burdock” OR “Arctii Fructus” OR “burdock root”) AND (“antidiabetic” OR “hypoglycemic” OR “AMPK” OR “GLUT4” OR “arctiin” OR “arctigenin” OR “inulin” OR “α-glucosidase” OR “streptozotocin” OR “alloxan” OR “HFD” OR “HbA1c” OR “HOMA-IR”). Studies were included if they provided original data on phytochemistry, molecular mechanisms, or traditional antidiabetic applications. Exclusion criteria included non-peer-reviewed abstracts and studies without full-text availability.

## 3. *A. lappa*: Botanical Review and Distribution

A detailed botanical characterization of *A. lappa* is essential for a diabetes-focused review as it directly impacts reproducibility and clinical translation. First, the concentration of key bioactive metabolites, such as inulin and lignans, varies significantly depending on the plant part used (roots, leaves, or seeds) [19]. Second, precise species identification is necessary to distinguish *A. lappa* from closely related species like *A. minus*, which may have different phytochemical profiles and biological activities [20]. Finally, establishing these parameters mitigates the risk of adulteration, ensuring that the therapeutic outcomes discussed are attributed to a verified and standardized botanical source.

**Figure 2:**
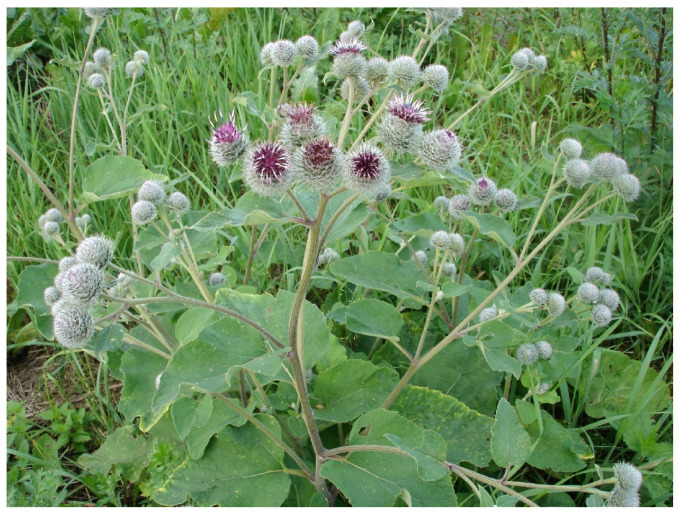
*Arctium lappa* from Kazakhstan. (Source: Original photograph by authors).

The species belongs to the Asteraceae family and the genus *Arctium*, which includes about 10 species distributed mainly in the temperate latitudes of Eurasia [33] (Figure 2). The plant was first scientifically described by Carl Linnaeus in 1753 in the fundamental work *Species Plantarum*, which became a key point for the classification and inclusion in *The Systematics of Angiosperms of the 18th Century*. It was during this period that the modern botanical nomenclature was actively formed, and burdock became one of the species that received an official Latin name and strict taxonomic characterization.

The systematic classification of *A. lappa* is given in Table 1 [34]. This species is a biennial herbaceous plant, reaching a height of 1.5–2 m. In the first year, a powerful taproot of up to 60 cm long and a rosette of basal leaves are formed, and in the second year, an erect, grooved, branched stem is formed [35]. The leaves are large and heart-shaped, with long petioles, dark green at the top, and grayish-pubescent below due to a thick coat of hair [19]. The inflorescences are spherical baskets up to 3–4 cm in diameter, collected at the ends of the branches. The flowers are tubular, purplish-purple, and bisexual, with a five-toothed corolla [21]. The wrapping of the baskets consists of many bracts with curved hook-shaped tips, to ensure attachment to clothing and animal hair, promoting the spread of seeds. The achenes are oblong, slightly curved, and topped with a deciduous pappus consisting of short, stiff bristles. Flowering occurs from July to September, and ripening happens in August–October. To ensure the reproducibility of pharmacological data, *A. lappa* must be distinguished from other closely related species often found in similar habitats, such as *A. minus* and *A. tomentosum*. Morphologically, *A. lappa* is characterized by its larger flower heads arranged in corymbose clusters and solid petioles, whereas *A. minus* possesses smaller heads (1.5–2.5 cm) in a more racemose arrangement and hollow petioles. *A. tomentosum* is easily identified by the dense, cobweb-like (tomentose) hairs covering its involucres [20]. Chemically, while these species share some common metabolites, *A. lappa* typically exhibits a higher concentration of the lignans arctiin and arctigenin, which are critical for its specific antihyperglycemic profile [20]. Recognizing these diagnostic features is vital to preventing the mixing of taxa in research and clinical applications.

**Figure 3:**
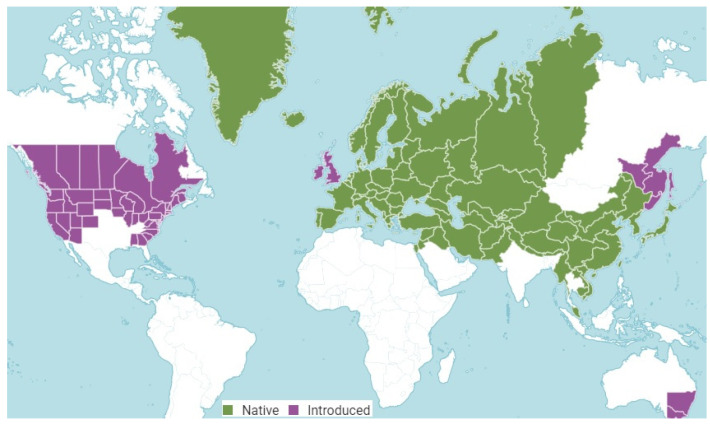
Distribution of *A. lappa* in the world. (Source [31]).

*A. lappa* is native to a vast Eurasian expanse, encompassing much of Europe (from Albania and Austria to Ukraine and the Baltic States), the Middle East and Central Asia (including Afghanistan, Iran, Iraq, Kazakhstan, and Türkiye), and East Asia (such as China, Japan, Republic of Korea, Mongolia, Nepal, Russia, and Siberia). It has been introduced widely elsewhere, notably across North America (e.g., from Alabama to Wyoming, including most Canadian provinces and U.S. states), the British Isles, parts of Russia (like Amur and Sakhalin), Australia (New South Wales, Victoria), and New Zealand [33] (Figure 3). The plant prefers moist, fertile, organic-rich soils and is found along forest edges, roads, on wastelands and riverbanks. In China and Japan, burdock is actively cultivated as a food and medicinal plant: its roots are eaten (known in Japan as gobo), and the seeds, leaves, and roots are included in the official pharmacopeias of these countries [29].

## 4. Traditional Uses of *A. lappa*

*A. lappa* boasts a long-documented history in traditional medicine for managing symptoms akin to diabetes or blood glucose dysregulation across diverse regions. However, a clear distinction must be made between its role as a functional food and its specialized medicinal applications. While the young, tender roots (known as “gobo”) and leaf stalks are widely consumed as dietary vegetables in Japanese and Korean traditions [30], these culinary uses differ from the concentrated therapeutic preparations found in pharmacopeias [19,36].

In traditional Chinese medicine (TCM), seeds known as Niubangzi were historically documented for “purifying” heat and toxins and were historically utilized in the management of inflammation and energy balance. Notably, 16th-century texts like *Ben Cao Gang Mu* described seed and root decoctions as remedies for “wasting-thirst syndrome” (xiaoke). In ethnopharmacological research, this condition is frequently treated as a historical correspondence hypothesis to modern DM, as symptoms like polydipsia align with the traditional description; however, this remains a traditional interpretation rather than a direct clinical equivalence [37]. In modern Chinese culture, the plant maintains a dual role; while crushed seeds relieve respiratory ailments, the roots are applied to systemic inflammatory conditions like gout and rheumatism, which often co-occur with metabolic syndrome [38].

From a regulatory perspective, the dried roots are the primary part officially recognized in various monographs, including the European Union herbal monograph and the State Pharmacopoeia of the Republic of Kazakhstan, for their metabolic and depurative properties. While the seeds (Fructus Arctii) are prominent in TCM, they are less commonly cited in Western pharmacopeias for diabetic indications [36,39].

Japanese and Korean traditions distinguish burdock roots, known as gobo, primarily as a food, yet deploy them therapeutically as a metabolic tonic. In Japan, roots are a common food crop, yet ethnobotanical records from the early Edo period in Fukushima Prefecture underscore their longstanding therapeutic use in large-quantity decoctions [30]. Tinctures and decoctions from roots were traditionally utilized with the intention of reducing blood sugar, alongside mild diuretic and hepatoprotective effects [19].

European phytotherapy has positioned burdock roots as a medicinal agent for glycemia disorders and obesity since the 19th century. In Southern Europe, applications vary from the Greek Central Macedonia region, where roots are consumed raw for detoxification and joint pain [40], to Central Italy (Molise), where stems are eaten raw before flowering, while root decoctions serve as depurative agents [41]. Even ritualistic uses appear in Albanian tradition, where the leaves are used in spiritual healing ceremonies [42]. Modern ethnobotanical surveys affirm root preparations for controlling glucose in patients with type 2 diabetes, often alongside liver and kidney support [19]. In Kazakhstan and Central Asia, root decoctions function in local tradition as a folk therapeutic for preventing what is colloquially termed “sugar disease” (a folk concept referring to symptoms of hyperglycemia), underscoring a blend of Eastern and European approaches to metabolic regulation [39,43]. Similarly, the traditional use of the root as a depurative agent is often described in folk contexts as a way to “purify the blood”: a concept that likely corresponds to the systemic elimination of metabolic waste products, although this lacks a direct biomedical equivalent. Furthermore, in the Indian Himalayan region, roots are recognized as a remedy for dermatological symptoms like eczema, which is frequently associated with chronic systemic inflammation in diabetic patients [44].

A comparative analysis of ethnomedical data (Table 2) demonstrates *A. lappa*’s broad therapeutic application against DM. This cross-cultural evidence provides a historical baseline, though it is important to note that pharmacopeial inclusion is generally restricted to standardized root preparations rather than the whole plant or its dietary forms.

**Table 2:** Ethnobotanical data on the use of *A. lappa* for DM.

Region/Country	Used Plant Part	Used Form and Method	Traditional Indication	References
China	Seeds, roots	Decoctions, infusions	“Clearing heat and thirst” (Xiaoke); metabolic symptoms	[40,45]
Japan	Roots (gobo)	Dietary use, Decoction	Blood sugar reduction; metabolic syndrome prevention	[43,45]
Republic of Korea	Roots, leaves	Infusion, Powder	Blood sugar control; hepatoprotection	[46]
India (Himalaya)	Roots	Folk remedy	Inflammation-related dermatological issues	[44]
Greece (Macedonia)	Roots	Raw, Decoction	Detoxification; anti-inflammatory support	[40]
Italy (Molise)	Roots, Stems	Decoction, Raw	Depurative effects; dietary metabolic support	[41]
Iran (Khuzestan)	Roots, leaves	Decoction, Infusion	Traditional management of diabetes	[47]
Serbia (Pirot district)	Roots, fruits	Tea, Infusion	Blood purification; traditional glucose control	[48]
Kazakhstan and Central Asia	Roots	Decoction, Infusion	Prevention of “sugar disease”; body cleansing	[49]
Türkiye (Anatolia)	Roots	Infusion	Antihyperglycemic and antioxidant activities	[50]
Bulgaria/Romania	Roots	Infusion, Extract	Glucose lowering; digestive support	[22,51]

## 5. Phytochemical Profile

### 5.1. Reported Compounds

The phytochemical profile of *A. lappa* is a rich and diverse database of biologically active compounds responsible for its broad pharmacological properties. This plant contains significant amounts of caffeoylquinic acid derivatives, flavonoids, lignans, and triterpenoids that play a key role in antioxidant and antihyperglycemic activity. Qualitative and quantitative composition varies depending on the plant part, vegetation stage, and extraction conditions [52,53,54,55,56,57].

Primary metabolism is characterized by carbohydrate compounds, which predominately include inulin, galactose, rhamnose, and fructans [24,54,58]. The roots are the primary source of inulin, typically containing between 30% and 50% by dry weight [19,59], representing a significant prebiotic and metabolic component. Secondary metabolites drive the plant’s specific pharmacological effects (Figure 1). Among the most researched are phenolic compounds: specifically, caffeoylquinic acid derivatives (including chlorogenic acid and cynarin) and flavonoids (quercetin, rutin, luteolin) [18,51,60]; lignans: notably, arctiin (5–9%) and arctigenin (0.6–1.1%) in the seeds, alongside diversas lappaols (A–F) [61,62]; terpenoids and sterols: triterpenes such as amyrin, lupeol, and phytosterols like sitosterol [38,56,57]; and polyacetylenes (Polyines): sulfur-containing derivatives like the thiophene arctinone B, and non-heterocyclic hydrocarbons like arctinol and arctinal [63].

### 5.2. Extraction and Analytical Characterization

The yield and stability of these compounds are highly dependent on the extraction technology used. Modern research utilizes a range of technologies, from traditional maceration to ultrasound-assisted extraction (UAE) and supercritical CO_2_ methods [55,56,57]. Aqueous and alcoholic solvents (primarily 50–70% ethanol) are used to isolate polar compounds such as phenolic acids and inulin [18,54,64]. To extract nonpolar compounds like terpenes and phytosterols, organic solvents or supercritical CO_2_ with ethanol as a co-solvent are preferred to increase selectivity [55,56,57].

Chemical characterization is conducted using integrated analytical platforms such as HPLC-DAD, UHPLC–MS/MS, GC-MS, and NMR spectroscopy [61,64]. These methods allow for detailed identification of structural features and functional groups (Table 3). While these innovative technologies aim to produce standardized extracts, the current literature often lacks rigorous standardization protocols and reproducible composition data. This absence of uniform quality control complicates direct comparisons between pharmacological studies and remains a primary barrier to clinical translation [58]. Based on current findings regarding yield efficiency and cost-effectiveness, UAE using aqueous ethanol (50–70%) is proposed as the most viable and economical method for producing standardized antidiabetic extracts. Furthermore, HPLC-DAD is recommended as the preferred analytical technique for routine quality control to ensure consistent concentrations of key markers such as arctigenin and chlorogenic acid in nutritional or pharmaceutical preparations.

**Table 3:** Phytochemical constituents of *A. lappa*, extraction methods, structural features (SAR), and experimental models for antihyperglycemic activity.

Compound Name	Plant Part	Extraction Method	SAR Insights	Study Model	Mechanism	Ref.
**LIGNANS (Subclass: Dibenzylbutyrolactones)**						
Arctiin	Seeds/Fruit	Hydroethanolic extraction; column chromatography	Lignan glycoside; serves as a precursor (prodrug) that requires conversion to arctigenin for maximum activity.	In vivo (STZ-induced diabetic rats)	Improves glucose tolerance; reduces fasting blood glucose; inhibits NF-κB.	[65]
Arctigenin	Seeds/Roots	Ethanol extraction; silica gel chromatography; HPLC	Dibenzylbutyrolactone skeleton; the methoxy groups and lactone ring are critical for activating the AMPK pathway.	In vivo (db/db mice); in vitro (HepG2 cells)	Potent AMPK activator; increases GLUT4 translocation; inhibits gluconeogenesis.	[60]
**LIGNANS (Subclass: Other Lignans/Furanofurans)**						
Lappaol A	Seeds	Methanol extraction	Sesquilignan structure	In vitro	Potent anti-inflammatory activity; inhibits NO production and pro-inflammatory cytokines (TNF-α and IL-6), which are key drivers of insulin resistance.	[66]
Lappaol C	Seeds	Methanol extraction	Complex lignin scaffold	In vitro	Exhibits strong antioxidant properties; protects hepatic cells from oxidative stress; and enhances glucose consumption in insulin-resistant cells.	[52]
Lappaol F	Seeds	Methanol extraction	Antioxidant phenolic groups	In vitro	Modulates the NF-κB signaling pathway; reduces cellular inflammation; and protects pancreatic β-cells from apoptosis (cell death).	[67]
Diarctigenin	Seeds	Methanol extraction	Dimeric form of arctigenin	In vitro	Shows higher potency than monomeric arctigenin in certain assays; significantly enhances glucose uptake in skeletal muscle and inhibits *PTP*1*B* (a negative regulator of insulin signaling).	[68]
**PHENOLIC ACIDS (Subclass: Caffeoylquinic Acids)**						
Chlorogenic acid	Leaves/Roots	Ultrasound-assisted extraction; aqueous solution/ethanol	An ester of caffeic acid and quinic acid; multiple vicinal hydroxyl (-OH) groups allow for high-affinity hydrogen bonding with α-glucosidase.	In vitro (enzymatic); In vivo (SHR rats)	Strong inhibition of α-glucosidase and α-amylase; reduces hepatic glucose output.	[52,53]
Caffeic acid	Roots	Methanol extraction; HPLC	Simple phenolic structure with a catechol group; the dihydroxy configuration is essential for potent antioxidant activity and protection of β-cells.	In vitro (cell-based); in vivo (STZ-rats)	Scavenges ROS; increases glucose uptake via GLUT4 translocation.	[69,70]
Cynarin (1,3-Dicaffeoylquinic acid)	Roots/Leaves	Aqueous/ethanol extraction	Dicaffeoylquinic acid isomer; the presence of two caffeoyl groups enhances its ability to inhibit glucose-6-phosphatase.	In vitro (hepatic microsomes)	Inhibits glucose-6-phosphate translocase; hepatoprotective effects.	[58,61,71]
1,5-Dicaffeoylquinic acid	Roots	Hydroethanolic extraction	Isomer of cynarin; the spatial orientation of the caffeoyl groups increases its inhibitory potency against aldose reductase.	In vitro (aldose reductase assay)	Inhibits aldose reductase; prevents diabetic complications like cataracts.	[72]
**CARBOHYDRATES/POLYSACCHARIDES (Subclass: Fructans)**						
Inulin	Roots	Hot water extraction; ethanol precipitation	High degree of polymerization (DP > 10) with β(2 ⟶ 1) glycosidic bonds; structural resistance to human digestion allows it to reach the colon intact.	In vivo (high-fat diet mice/clinical)	Acts as a prebiotic; increases GLP-1 secretion; improves insulin sensitivity and gut microbiota composition.	[59,61,73]
Inulooligosaccharides	Roots	Enzymatic hydrolysis of inulin; water extraction	Shorter chain length (DP 3–10); higher solubility than inulin. The specific β-linkages promote the growth of *Bifidobacterium.*	In vitro (fermentation model); in vivo (rat)	Stimulates short-chain fatty acid (SCFA) production; lowers postprandial glucose.	[74]
Burdock Fructooligosaccharides (BFOs)	Roots	Aqueous extraction; Membrane filtration	A complex mixture of fructooligosaccharides unique to *A. lappa*; the branching and chain length distribution are key to its antihyperglycemic potency.	In vivo (STZ-induced diabetic mice)	Protects islet β-cells from oxidative stress; regulates hepatic glycogen synthesis.	[75,76]
**TERPENOIDS (Subclass: Sesquiterpenes/Guaianolides)**						
Dehydrocostus lactone	Roots	Methanol extraction; Supercritical CO_2_ extraction	Sesquiterpene lactone; contains an exocyclic double bond that reacts with thiol groups in proteins (Michael addition), enhancing its anti-inflammatory and insulin-sensitizing effects.	In vitro (adipocytes); in vivo (mice)	Inhibits NF-κB signaling; improves insulin sensitivity; reduces adipocyte differentiation.	[77,78]
Costunolide	Roots/Leaves	Ethanol extraction; Steam distillation	Germacranolide structure; the α-methyleneγ-lactone moiety is essential for its ability to induce antioxidant enzymes via the Nrf2 pathway.	In vivo (STZ-rats); in vitro (cell-based)	Protects β-cells from oxidative damage; promotes glucose-stimulated insulin secretion (GSIS).	[79,80]
**SULFUR-CONTAINING COMPOUNDS (Subclass: Polyynes/Thiophenes)**						
Arctinol	Roots	Methanol/Ethyl acetate extraction; Column chromatography	Thiophene derivative with an alcohol side chain; the sulfur atom in the heterocyclic ring enhances its ability to stabilize free radicals and inhibit oxidative damage.	In vitro (DPPH/ABTS assays)	Potent antioxidant; protects pancreatic β-cells from oxidative stress-induced apoptosis.	[81,82]
Arctinal	Roots	Methanol extraction	Thiophene derivative with an aldehyde group; the electrophilic nature of the aldehyde group allows it to interact with specific enzymatic residues, potentially inhibiting carbohydrate-digesting enzymes.	In vitro (enzymatic)	Antioxidant; moderate α-glucosidase inhibition; antimicrobial activity.	[63]
Sulfur-containing acetylenes	Roots	Petroleum ether or Hexane extraction; Silica gel chromatography	Characterized by a chain of conjugated triple bonds with sulfur substitutions; the high degree of unsaturation makes them potent lipophilic antioxidants that protect cell membranes.	In vitro (cellular assays)	Reduces lipid peroxidation; modulates glucose transporters; antimicrobial activity.	[81,83]
**LIPIDS (Subclass: Fatty Acids)**						
Linoleic acid	Seeds	Hexane extraction; Soxhlet extraction	Polyunsaturated fatty acid (PUFA) with two double bonds; crucial for maintaining cell membrane fluidity and acts as a precursor to anti-inflammatory eicosanoids.	In vivo (animal models)	Improves insulin sensitivity; reduces chronic low-grade inflammation associated with obesity.	[38]
Oleic acid	Seeds	Cold pressing; Solvent extraction	Monounsaturated fatty acid (MUFA) with one double bond; provides energy and supports metabolic health by reducing oxidative stress in adipocytes.	In vivo (animal models)	Enhances glucose uptake in peripheral tissues; protects against lipotoxicity.	[38]
Palmitic acid	Seeds	Soxhlet extraction	Saturated fatty acid; provides structural stability to cell membranes, although excess levels can be linked to insulin resistance.	In vitro/in vivo	Component of cell membranes; studies often focus on its role in metabolic regulation.	[38,84]
Pentadecanal	Roots/Leaves	Solvent extraction	Saturated long-chain aldehyde (C_15_H_30_O); contributes to the hydrophobic antioxidant capacity of the lipid fraction.	In vitro	Antioxidant activity; stabilizes lipid membranes against peroxidation.	[57]
**OTHER COMPOUNDS (Subclass: Flavonoids/Sterols)**						
Quercetin	Leaves/Flowers	Hydroethanolic extraction; Column chromatography	Pentahydroxyflavone; the 3′, 4′-dihydroxy groups on the B-ring and the 3-OH group on the C-ring are essential for inhibiting glucose-6-phosphatase.	In vitro; in vivo (STZ mice)	Enhances insulin secretion; inhibits α-glucosidase; reduces gluconeogenesis.	[52,53,85]
Rutin	Leaves	Aqueous/Methanol extraction	Glycoside form of quercetin (quercetin-3-O-rutinoside); the sugar moiety improves water solubility and bioavailability in the gut compared to the aglycone.	In vivo (HFD-rats)	Antioxidant; protects against diabetic nephropathy; improves glucose uptake.	[52,53,86]
β-sitosterol	Roots/Seeds	Chloroform or Hexane extraction	Phytosterol with a 4-desmethyl sterol skeleton; its structure is similar to cholesterol, allowing it to compete for absorption and modulate membrane-bound transporters.	In vivo (diabetic rats)	Lowers cholesterol; improves fasting blood glucose; stimulates insulin secretion.	[55,56]

## 6. Pharmacological Evidence of Antihyperglycemic Activity

Among the widely studied bioactivities of the plant, a particular function of interest is the plant’s ability to exert antihyperglycemic and antidiabetic effects, which makes it a promising source of natural remedies for correcting carbohydrate metabolism disorders. Numerous experimental data indicate that the biological effects of burdock are realized through a combination of antioxidant, enzyme-inhibiting, anti-inflammatory, and insulin-modulating mechanisms [87,88,89].

One of the main directions of pharmacological study of *A. lappa* is the assessment of its ability to inhibit key enzymes of carbohydrate metabolism (α-amylase and α-glucosidase). These enzymes catalyze the hydrolysis of complex carbohydrates to monosaccharides, and their suppression helps to reduce the postprandial increase in blood glucose levels. Extracts of the leaves and roots of *A. lappa*, rich in phenolic acids (chlorogenic, caffeic, and dicaffeoylquinic) and flavonoids (rutin, quercetin, and luteolin), exhibit pronounced inhibitory activity against yeast-derived α-glucosidase (IC_50_ ≈ 45.2 µg /mL) and porcine α-amylase (IC_50_ ≈ 62.5 µg/mL). These effects are statistically non-inferior to acarbose (IC_50_ ≈ 38.4 µg/mL) under the same assay conditions, demonstrating high binding affinity to the enzyme catalytic sites [64]. It is assumed that chlorogenic and cynarine acids bind to the active center of the enzyme through hydrogen bonds, preventing the breakdown of oligosaccharides. Flavonoids, due to their polyphenolic structure, are able to block the catalytic sites of α-amylase, thereby reducing the rate of starch hydrolysis [64,90]. Mechanistic studies show that the lignans arctigenin and arctiin are also involved in the inhibition of α-glucosidase and can alter the conformation of the enzyme, acting as non-competitive inhibitors. This mechanism is associated with the presence of aromatic hydroxyl groups responsible for complexation with amino acid residues of the active site [62,91]. Thus, in vitro enzymatic studies confirm that phenolic and lignan compounds of *A. lappa* have a pronounced ability to slow down carbohydrate hydrolysis and reduce glycemic load.

Pharmacological experiments on animal models of diabetes have demonstrated the stable antihyperglycemic activity of *A. lappa* extracts, especially in relation to the regulation of glycemia and lipid profile. In experiments with rats in which diabetes was induced by streptozotocin (STZ) or alloxan, the administration of hydroethanolic and methanol burdock extracts (200 mg/kg/day for 28 days) led to a significant decrease in blood glucose levels, an improvement in body weight, and a decrease in triglyceride concentrations [87,89]. The mechanisms of the observed action are associated with the multifactorial influence of a complex of plant compounds. Phenolic acids and flavonoids increase tissue sensitivity to insulin, protecting pancreatic beta cells from oxidative stress and apoptosis. Polysaccharides and inulin contribute to the normalization of lipid metabolism by regulating PKC/NF-kB and SREBP-1/SCD-1 signaling pathways [29,92]. Particular attention is paid to the lignan arctigenin, which is converted into arctigenic acid in the body, a compound that stimulates early insulin secretion and restores impaired glucose tolerance [88]. In addition, total burdock seed lignans have demonstrated a pronounced preventive effect in diabetic retinopathy, preventing damage to the vascular retina [29]. These results confirm the involvement of *A. lappa* compounds in maintaining metabolic homeostasis and regulating endocrine functions. Also, it was noted that the water-soluble polysaccharides of *A. lappa* roots have antioxidant and anti-inflammatory effects, reducing the level of pro-inflammatory cytokines and inhibiting the activity of NO synthase in pancreatic tissues [92,93]. This allows us to consider these extracts as functional biological products with a combined antihyperglycemic and cytoprotective effect.

Despite the limited number of clinical trials, the results of available studies demonstrate the promise of using *A. lappa* in the treatment of metabolic disorders in humans. Beyond preclinical models, the metabolic benefits of *A. lappa* have been observed in clinical settings. Elderly women with metabolic syndrome demonstrated that supplementation with burdock root extract significantly improved abdominal obesity and modulated sex hormone levels. These findings suggest that the plant may serve as a valuable functional food or adjunct therapy for managing a cluster of metabolic disturbances that often precede or accompany type 2 diabetes [32]. However, while these results are promising for general metabolic health, targeted randomized controlled trials focusing specifically on glycemic endpoints like HbA1c are lacking in the current literature. Additional observations in experimental models show that regular use of *A. lappa* extracts leads to significant improvements in glycemic control and lipid metabolism. These effects are attributed to the synergistic action of bioavailable flavonoids and lignans [87], though further clinical trials are needed to confirm these results in human populations. In East Asian countries, the plant is traditionally used in the diet of people with type 2 diabetes in the form of decoctions and food additives, and numerous ethnopharmacological data confirm its antihyperglycemic potential [23]. Nevertheless, for a final assessment of the clinical efficacy of *A. lappa*, randomized, placebo-controlled trials should be conducted, taking into account dose dependence, standardization of extracts, and pharmacokinetic parameters of active substances. The development of standardized extracts with a certain content of arctigenin, chlorogenic acid and inulin can increase the reproducibility of pharmacological effects and create a scientifically sound basis for the use of *A. lappa* in the medical phytotherapy of diabetes and metabolic syndrome.

A combination of experimental and clinical data indicates that *A. lappa* has pronounced antihyperglycemic activity, realized through a combination of antioxidant, enzyme-inhibiting, anti-inflammatory and insulin-regulating mechanisms. The multifactorial action of the phytocomplex makes the plant a promising source of natural compounds for the development of antidiabetic phytopreparations and functional foods aimed at the prevention and correction of metabolic disorders. A brief summary of the pharmacological effects and mechanisms of action in extracts of *A. lappa* is presented in Table 4 [28]. Despite the promising results observed in the cited studies, several limitations must be addressed. Most pharmacological evidence for the antihyperglycemic effects of *A. lappa* is derived from in vitro assays or acute animal models (e.g., STZ-induced diabetic rats as a model of insulin-deficient type 1-like diabetes). There is a notable lack of long-term, double-blind, placebo-controlled human clinical trials. Additionally, the high variability in extraction methods and the absence of standardized dosage protocols across the literature make it difficult to establish a definitive therapeutic window. Future research must focus on standardized clinical evaluations to confirm the safety and efficacy of these extracts in human populations.

**Table 4:** Antidiabetic effects of *A. lappa* (in vitro/in vivo models).

Model/Study Type	Plant Part/Form Used	Dose/Duration	Key Mechanisms of Action	Pharmacological Effect	References
In vitro (enzyme inhibition)	Root extract	100–1000 µg/mL	Inhibition of α-glucosidase and α-amylase	Reduction in postprandial hyperglycemia	[22,27,28]
In vitro (antioxidant tests: DPPH, ABTS)	Polysaccharides/fructans (roots)	0.5–5 mg/mL	↓ ROS; ↑ activity of antioxidant enzymes (SOD, CAT, GPx)	Antioxidant protection of β-cells, cytoprotection	[24,45,54]
In vitro (adipocytes 3T3-L1)	Arctigenin/arctiin (lignans)	10–50 µM	Modulation of PPAR-γ; stimulation of lipolysis	Reduction in adipogenesis, improvement of lipid metabolism	[90,91,94]
In vitro (β-cell culture/oxidative stress model)	Dicaffeoylquinic acids	25–100 µM	Protection against oxidative stress; stabilization of β-cells	Improved insulin secretion	[61,64,73]
In vivo (STZ (T1DM-like))	Aqueous/alcoholic root extract	200–400 mg/kg (28 days)	↑ Insulin sensitivity; ↑ GLUT4 expression	Lower fasting glucose levels	[28,95]
In vivo (type 2 diabetes model)	Ethanolic leaf extract	150–300 mg/kg (21–28 days)	Suppression of NF-κB; ↓ TNF-α	Anti-inflammatory effect, improved glycemia	[55,90]
In vivo (obesity + insulin resistance)	Arctigenin (pure compound)	10–20 mg/kg (4–6 weeks)	Activation of AMPK; reduced lipogenesis	Improved glucose utilization, decreased insulin resistance	[91,94]
In vivo (liver protection in diabetes)	Fructans/inulin (roots)	100 mg/kg (28 days)	↑ Antioxidant activity of liver; ↓ oxidative stress markers	Hepatoprotective effect	[24,96]
In vivo (lipid profile correction)	Seed extract	200 mg/kg (28 days)	Regulation of lipid metabolism (↓ LDL, ↑ HDL)	Reduced risk of dyslipidemia-related complications	[51,62]
In vivo (neuroprotection)	Lignans (arctigenin/arctiin)	5–10 mg/kg (14–21 days)	↓ NO production; suppression of inflammation; antioxidant activity	Neuroprotective effect in diabetic complications	[63]

The pharmacokinetics of *A. lappa* lignans involve significant biotransformation by the gut microbiota. Specifically, the glycoside arctiin is hydrolyzed into its aglycone form, arctigenin, which represents the primary bioavailable active metabolite. Subsequent hepatic or microbial metabolism may further convert arctigenin into arctigenic acid or various glucuronide and sulfate conjugates. However, current evidence indicates that arctigenin itself is the principal agent responsible for systemic pharmacological effects, such as AMPK activation and GLUT4 translocation, while further metabolites like arctigenic acid are likely secondary or inactive.

## 7. Molecular Mechanisms Underlying Antihyperglycemic Action

The pharmacological effect of *A. lappa* is determined by the multicomponent action of its phytocomplex and is realized through several overlapping molecular mechanisms: modulation of insulin secretion and sensitivity to it, regulation of glucose utilization and transport pathways (including GLUT4 translocation and AMPK activation), and indirect effects, antioxidant, anti-inflammatory and hepatoprotective activity, contributing to the maintenance of glycemic homeostasis. A summary of the key compounds, their molecular mechanisms and pharmacological effects is given in Table 5.

**Table 5:** Major chemical compounds, their molecular mechanisms, and antidiabetic-related pharmacological effects of *A. lappa*.

Compound/Group	Molecular Mechanisms	Pharmacological Effect (Antidiabetic + Related Effects)	Model	Ref.
**AXIS 1: Enzyme Inhibition**				
Chlorogenic acid	Inhibition of α-glucosidase; antioxidant activity	Antihyperglycemic effect; protection of β-cells	In vitro (phenolic profiling and antioxidant-related evaluation)	[52,53,54]
General phenolic complex	Inhibition of carbohydrate hydrolases (α-glucosidase/α-amylase)	Overall antihyperglycemic and cytoprotective effects	In vitro (phenolic profiling and antioxidant assays)	[45,52,53,54]
**AXIS 2: Antioxidant and Anti-inflammatory**				
Dicaffeoylquinic acids (including cynarin)	Protection of β-cells; strong antioxidant and anti-inflammatory activity	Reduction in serum glucose; improvement of insulin secretion	In vitro (SAR/free radical scavenging assays; compound isolation and analysis)	[61,64,73]
Polysaccharides (roots)	↓ ROS; ↑ activity of antioxidant enzymes (SOD, CAT, GPx)	Hepatoprotective and antioxidant effects in diabetes	In vitro + in vivo (antioxidant assays; animal models)	[24,45]
Quercetin and its derivatives	Antioxidant; membrane stabilization	Reduction in oxidative stress; cytoprotection	In vitro (phenolic profiling; antioxidant evaluation)	[52,53]
Caffeic acid	Antioxidant; modulation of carbohydrate-metabolizing enzymes	Antihyperglycemic activity; improvement of glucose metabolism	In vitro (phenolic profiling/UPLC-MS identification)	[52,53]
Sulfur-containing polyacetylenes	Antioxidant and anti-inflammatory mechanisms	Potential benefit in inflammatory metabolic disturbances	In vitro (compound isolation and structural identification)	[63]
**AXIS 3: AMPK and Insulin Signaling**				
Arctigenin (lignan)	Associated with AMPK signaling pathways; suppression of NF-κB; ↓ NO production	Improved insulin sensitivity; anti-inflammatory and neuroprotective effects	In vitro + in vivo (cell models; NO inhibition; antihyperglycemic animal models)	[88,91,94]
Arctiin (lignan)	Downregulation of inflammatory markers (e.g., NO, cytokines)	Tissue protection; anti-inflammatory activity	In vitro + in vivo (NO inhibition assay; C. elegans model)	[62,90]
**AXIS 4: Gut Microbiota and Prebiotic**				
Fructans/inulin	Observed shifts in gut microbiota composition	Mild antihyperglycemic effect; prebiotic activity	In vitro + in vivo (antioxidant assays; animal experiments)	[24,59]

The antidiabetic-related activity of *A. lappa* compounds is strongly associated with their structural features. Phenolic acids such as chlorogenic and caffeic acids exhibit antioxidant and enzyme-inhibitory potential due to the presence of hydroxyl groups (−OH) and conjugated aromatic rings, which enhance radical scavenging capacity and may contribute to glucose metabolism regulation. Dicaffeoylquinic acid derivatives demonstrate stronger antioxidant effects than monocaffeoyl derivatives, as the presence of two caffeoyl moieties increases electron-donating ability and stabilizes reactive intermediates. Flavonoids such as quercetin possess a planar polyphenolic scaffold with multiple hydroxyl substituents, which correlates with ROS neutralization and membrane-protective effects. Lignans (arctigenin/arctiin), being more lipophilic, can interact with intracellular signaling pathways (e.g., AMPK activation and NF-κB suppression), which explains their anti-inflammatory and insulin-sensitizing properties. High-molecular-weight carbohydrates (fructans, inulin, and polysaccharides) mainly act indirectly through prebiotic mechanisms and enhancement of antioxidant enzyme systems rather than direct receptor interactions.

Understanding the cumulative effect of the *A. lappa* phytocomplex makes it possible to identify key application points of its biologically active substances, each of which contributes to maintaining glycemic balance. One of the central directions of such an effect is the regulation of the work of beta-cells of the pancreas and an increase in the effectiveness of insulin-dependent mechanisms of glucose utilization. It is these processes that underlie the first block of mechanisms of antihyperglycemic action in the plant, such as the modulation of insulin secretion and insulin sensitivity. According to a meta-analysis of 16 experimental studies, it was shown that *A. lappa* significantly lowers fasting blood glucose, with a large standardized mean difference (SMD = −1.42). However, considerable heterogeneity (I^2^ > 75%) was observed, likely due to variations in extract preparation, dosage, and intervention duration [21].

**Figure 4:**
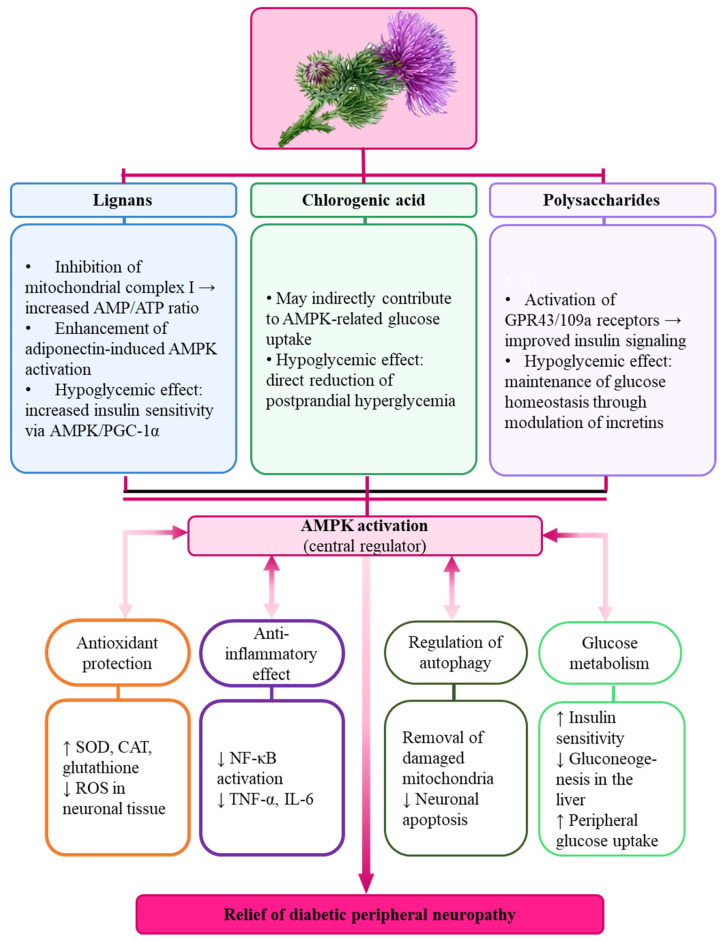
Proposed schematic representation of AMPK-mediated mechanisms of antihyperglycemic and neuroprotective effects of major *A. lappa* constituents (lignans, chlorogenic acid, and polysaccharides) in diabetic peripheral neuropathy. (Source: Original illustration prepared by authors based on the discussed pathways).

Chlorogenic acid and lignans from Arctii Fructus, specifically arctigenin, stimulate AMPK via two key mechanisms: inhibition of mitochondrial complex I (typically observed at concentrations of 1–10 µM), leading to an increased AMP/ATP ratio (metformin-like metabolic signaling), and enhancement of adiponectin-induced AMPK activation. While these micromolar concentrations are well-documented in vitro, it is important to note that systemic peak plasma levels (C_max_) after oral intake generally remain in the low nanomolar range (approx. 10–50 nM). This suggests that inhibition of direct complex I may be most physiologically relevant in high-exposure tissues like the intestinal mucosa and liver, whereas systemic effects likely involve additional signaling pathways. Activated AMPK acts as a central metabolic regulator coordinating glucose metabolism, oxidative stress responses, inflammation, and autophagy, which are critically involved in the development of diabetic complications. As summarized in Figure 4, AMPK activation enhances antioxidant defense by reducing ROS levels and increasing SOD, CAT, and glutathione; it suppresses inflammatory mediators via reduced NF-κB activation and decreased TNF-α and IL-6; promotes ULK1-related autophagy and cellular clearance; and improves glucose metabolism by increasing insulin sensitivity, reducing hepatic gluconeogenesis, and enhancing peripheral glucose uptake (Figure 4).

In in vitro studies, high concentrations of chlorogenic acid (up to 2 mmol/L) significantly increase glucose uptake in skeletal muscle tissue. However, from a translational perspective, such concentrations are not achievable in human plasma through oral intake, where levels typically peak in the low micromolar range (µM). Consequently, while these findings provide insight into the compound’s molecular potential, their direct physiological relevance to systemic glucose regulation in humans is limited and likely represents a pharmacological rather than a nutritional effect [97]. These data are consistent with the results of other studies demonstrating the role of *A. lappa* phenolic compounds (in particular, arctigenin) in stimulating insulin secretion, protecting beta cells, and increasing GLP-1 levels.

The enhancement of peripheral glucose uptake by *A. lappa* appears to involve two distinct signaling axes. First, compounds such as chlorogenic acid have been associated with the insulin-dependent pathway, potentially modulating IRS-1 phosphorylation and PI3K/AKT signaling. Second, and perhaps more prominently, arctigenin activates the insulin-independent AMPK pathway. While both axes ultimately promote GLUT4 translocation, the evidence for direct PI3K activation remains largely preliminary (hypothesis-level), whereas the AMPK-mediated mechanism is more robustly supported in current experimental models. This modulation of molecular cascades creates favorable conditions for the correction of hyperglycemia. The high content of phenolic acids and flavonoids gives burdock extracts significant antioxidant properties. These compounds reduce oxidative stress in β-cells, suppress NF-kB-mediated expression of proinflammatory cytokines, and reduce liver lipotoxicity [98]. This improves insulin sensitivity and slows down the progression of insulin resistance. Water-soluble polysaccharides of *A. lappa*, including inulin and fructans, have a prebiotic effect: they normalize the intestinal microbiota and reduce the level of systemic inflammation, which additionally supports glycemic control [99]. The totality of experimental data confirms a multi-level synergistic effect: direct stimulation of insulin secretion and regulation of key metabolic pathways (IRS/GLUT4, AMPK), combined with the antioxidant, anti-inflammatory and prebiotic effects of polysaccharides, form the complex antihyperglycemic potential of *A. lappa*.

Given the low systemic bioavailability of parent lignans like arctigenin, the physiological plausibility of direct systemic AMPK activation requires careful consideration. It is increasingly likely that dominant antidiabetic mechanisms are localized or mediated. These include intestinal effects, where high local concentrations of lignans and phenolics inhibit carbohydrate hydrolases [45,52,53,54]; microbiota-mediated effects, where gut bacteria transform arctiin into bioavailable arctigenin or produce Short-Chain Fatty Acids (SCFAs) that signal systemically [24,99]; and active metabolites, such as glucuronide conjugates, which may possess signaling properties distinct from the parent compound [88]. Consequently, *A. lappa* may function more as a “prodrug” or a gut-targeted intervention rather than a traditional systemic agent.

## 8. Safety, Toxicity and Dosage Considerations

Different studies show that *A. lappa* extracts generally have low acute toxicity, though this varies by preparation method and dose, with purified lignans like arctigenin posing higher risks than complex extracts. Gut metabolism sharply limits oral bioavailability, while accumulation in the heart, liver, and kidneys drives organ-specific effects after repeated dosing [100,101,102]. Importantly, these toxicological findings often involve related species such as *A. tomentosum* or purified lignans, which may not directly reflect the safety profile of traditional *A. lappa* root preparations. It is critical to note that toxicological thresholds observed in animal models (e.g., rats and dogs) do not translate directly to humans due to significant differences in metabolic rates and body surface area. Traditional use employs far lower amounts than preclinical thresholds. According to pharmacopoeial standards [36], the typical dosage for aqueous decoctions of the dried root or fruit ranges significantly depending on the clinical indication. These values should be viewed as broad traditional guidelines rather than precise clinical prescriptions, as therapeutic outcomes depend heavily on the extraction efficiency and the specific plant part used. Safety is highly dependent on the preparation method; for instance, aqueous decoctions of the dried root typically present a lower risk profile than concentrated ethanolic extracts. Furthermore, the toxicological profile varies significantly based on the plant part (root vs. fruit/seed) and the specific marker content, particularly the concentration of arctigenin, which should be standardized to ensure safety. To ensure clinical safety, animal dosages should be converted to a HED; for example, a dose of 250 mg/kg for a rodent scales to approximately 40 mg/kg in humans, or roughly 2.8 g for a 70 kg adult, which aligns with the upper limits of traditional preparation ranges. However, this HED serves as an illustrative conversion for toxicological comparison rather than a clinically validated dosage; extract standardization remains a prerequisite for these benchmarks to be clinically meaningful. Oral routes remain safest, with precautions needed for pregnancy and Asteraceae allergies. Regarding herb–drug interactions, practical clinical caution is warranted for patients currently utilizing antidiabetic medications (e.g., metformin or sulfonylureas). While specific biochemical interaction mechanisms remain unestablished in humans, the additive glucose-lowering effects of *A. lappa* could theoretically increase the risk of hypoglycemia. Therefore, co-administration requires diligent blood glucose monitoring, although further clinical research is needed to determine the actual incidence and severity of such interactions. Monitoring liver, kidney, and heart function also makes sense for long-term use. It is important to clarify that certain pharmacokinetic benchmarks were derived from subcutaneous administration to determine maximum systemic exposure; however, these results have limited direct relevance to the oral route recommended in this review. Oral intake involves first-pass metabolism and intestinal degradation, which significantly reduces systemic toxicity compared to parenteral routes. The key toxicological and pharmacological benchmarks are summarized in Table 6. Data regarding acute and subchronic toxicity in rodents [101] represent primary experimental outcomes, while the HED and safety thresholds for purified lignans [102] are aggregated values synthesized from multiple toxicological reports.

**Table 6:** Summary of toxicity, HED, and dosage data for *A. lappa*.

Parameter	Key Findings	Dose/Values	Route/Model	Source Type	Ref.
Acute Oral LD_50_	Low acute toxicity	>2000–5000 mg/kg	Oral, Rodents	Primary Study	[101]
Arctigenin NOAEL	High risk (non-oral)	<6 mg/kg/day	Subcutaneous/Parenteral, Dogs (*A. tomentosum*)	Primary Study	[100]
Oral LOAEL	Myocardial/renal damage	12–36 mg/kg	Oral, Rats (*A. tomentosum*)	Primary Study	[100,101]
ALFE Extract	Ethanolic fruit (seed) extract	LD_50_ > 5000 mg/kg	Oral, Rats	Primary Study	[101]
HED Estimate	Illustrative calculation only	~2.8 g/day	Extrapolated for 70 kg Adults	Review/Estimate	[102]
Traditional Dose	Aqueous decoction (dried root)	Variable traditional ranges	Oral, Human	Pharmacopeial Reference (EMA)	[36]

## 9. Conclusions

The current body of evidence suggests that *A. lappa* is a potential natural source of biologically active compounds, though its clinical antihyperglycemic efficacy is not yet established. While phenolic acids, flavonoids, lignans, and inulin contribute to metabolic modulation in experimental models, it must be emphasized that the vast majority of these findings are derived from preclinical data. Consequently, the significant antihyperglycemic potential observed in vitro and in animals remains to be validated through rigorous human trials. Alongside direct glycemic regulation, extracts of *A. lappa* provide antioxidant, anti-inflammatory and hepatoprotective effects, supporting the prevention of diabetes-related complications. Prebiotic polysaccharides further strengthen metabolic balance by influencing the gut microbiota and reinforcing the plant’s multifaceted biological action. Despite these advantages, this review highlights several limitations. Most pharmacological data originate from in vitro experiments and animal models, which restrict direct extrapolation to clinical settings.

Specifically, a critical clinical evidence gap remains characterized by a notable absence of randomized controlled trials (RCTs) in diabetic populations that utilize primary glycemic endpoints, such as HbA1c and fasting blood glucose levels, with standardized *A. lappa* products. Currently, most human data are limited to non-diabetic cohorts or secondary metabolic markers, meaning that established clinical efficacy for glycemic control remains speculative. The phytochemical composition of *A. lappa* varies markedly depending on extraction methods, plant part, geographical origin and standardization protocols, complicating cross-study comparisons. Clinical research remains scarce and is characterized by small sample sizes, limited randomization, insufficient placebo control and a lack of pharmacokinetic evaluation. Furthermore, long-term safety data are limited, particularly regarding the cumulative toxicity of lignans such as arctigenin.

These constraints underline the necessity for more rigorous scientific work. Specifically, future research should prioritize high-quality randomized controlled clinical trials using standardized extracts; detailed human pharmacokinetic studies to determine bioavailability; long-term safety and cumulative toxicity assessments of lignans; and investigation of the synergistic interaction between lignans and the gut microbiota. Such directions will help translate preclinical findings into effective and evidence-based therapeutic applications. Furthermore, a clear translational distinction must be maintained when evaluating the plant’s potential as a functional food where its fiber and inulin provide broad metabolic support; as a standardized extract acting as a therapeutic adjunct; and as a source of isolated lignans (e.g., arctigenin) serving as a drug leading to new pharmacotherapies. Each of these roles carries distinct thresholds and safety expectations. Overall, *A. lappa* represents a candidate for further investigation into safe and effective strategies within modern metabolic health frameworks.

## Figures and Tables

**Table 1:** Classification of *A. lappa*.

Kingdom	Plantae
Phylum	Tracheophyta
Class	Magnoliopsida
Order	Asterales
Family	Asteraceae
Genus	*Arctium* L.
Species	*Arctium lappa* L.

## Data Availability

No new data were created or analyzed in this study.
